# The Importance of Spirituality for Women Facing Breast Cancer Diagnosis: A Qualitative Study

**DOI:** 10.3390/ijerph18126415

**Published:** 2021-06-13

**Authors:** Diva Cristina Morett Romano Leão, Eliane Ramos Pereira, María Nieves Pérez-Marfil, Rose Mary Costa Rosa Andrade Silva, Angelo Braga Mendonça, Renata Carla Nencetti Pereira Rocha, María Paz García-Caro

**Affiliations:** 1 Faculty of Nursing, Fluminense Federal University, Niterói, Rio de Janeiro 24020091, Brazil; elianeramos.uff@gmail.com (E.R.P.); roserosa.uff@gmail.com (R.M.C.R.A.S.); rnencetti@yahoo.com.br (R.C.N.P.R.); 2 Department of Nursing, Mind Brain and Behavior Research Center (CIMCYC), University of Granada, 18071 Granada, Spain; mpazgc@ugr.es; 3 Department of Personality, Evaluation, and Psychological Treatment, Mind, Brain and Behavior Research Center, University of Granada, 18071 Granada, Spain; nperez@ugr.es; 4 National Cancer Institute, Rio de Janeiro 20230130, Brazil; angeloprimax@gmail.com

**Keywords:** breast cancer, spirituality, diagnosis, health, nursing, meaning in life, qualitative research

## Abstract

Breast cancer remains significantly distressing and produces profound changes in women’s lives. Spirituality is an important resource at the time of diagnosis and treatment decisions. This qualitative study aimed to explore the spiritual experience of women diagnosed with breast cancer and the considerations of spirituality in health care using the existential phenomenology approach. The sampling procedure was intentional, based on the study’s exclusion and inclusion criteria. Forty women participated in individual interviews. The research was conducted in the outpatient clinic of a reference federal university hospital in South-Eastern Brazil. Throughout the research process, ethical principles were carefully followed. Five themes were identified: (1) meaning of spirituality–source of spiritual strength, (2) well-being in the relationship with God, (3) well-being in religious fellowship, (4) values and purpose of life–meaning in life, and (5) spirituality as a foundation to continue. Respect for patient’s spiritual values was recognised as a fundamental principle in health care. Spirituality was revealed as a source of support during the complex process of being diagnosed with breast cancer. Thus, health care professionals that value and encourage spirituality are needed, favouring better patient response to the diagnosis.

## 1. Introduction

The diagnosis of breast cancer has proven to be challenging for all affected women, causing different levels of emotional distress [1,2,3,4]. The stigma associated with breast cancer requires patients to comprehend new concepts while experiencing helplessness, anxiety, depression, and meaninglessness, all in the face of suffering and uncertainty about the life that the disease and its treatment have created for them [5,6,7]. Despite improvements in diagnosis and treatment, breast cancer remains a significantly stressful event, producing psychological distress that can remain elevated for up to a year post-diagnosis. These effects should be recognised because they significantly impact women’s lives negatively [8,9,10,11,12].

In recent decades, scientific contributions to the spiritual dimensions of health have been increasing [1,8,13,14,15]. Spirituality is now seen as an important dimension of health and well-being, and, alongside religious faith, many individuals from diverse cultures may rely on it when confronted with severe illnesses such as cancer [8,16,17]. Spirituality refers to a set of emotions and non-material convictions [18] and must be understood as a search for connection with oneself, achieving a state of wholeness, a connection with others and the environment, and a personal connection with the sacred in an experience with the transcendent [19,20]. Moreover, spirituality promotes healing; incorporates values, attitudes, and perspectives to life; and leads to awareness of the meaning and purpose of life. Spirituality is connected to religious faith, but it is broader than this. Religiosity is an expression of spirituality, that in turn is deeply inserted into organised systems of religion or faith, including beliefs, practices, and religious rituals for a substantial experience. The spiritual dimension, which is focused on the integration of the physical, emotional, and spiritual, is valued as unique [15,20,21]. For instance, in women’s confrontations with breast cancer, they begin to explore existential questions such as those related to the search for meaning in life and transcendence. This can result in a greater will to live and can offer them comfort and hope in the face of distress and fear of finitude. [14,21].

There is sufficient support for the notion that spiritual factors can operate as a resource for individuals facing a cancer diagnosis and treatment decisions. Typically, women with breast cancer engage in positive coping strategies (which can be used to provide security and peace, obtain comfort, will to live, hope, and promote well-being), but it is worth mentioning that negative spiritual coping exists (e.g., feeling abandoned by God, distress, and negative emotions like high anxiety and acute fear) [5,8,14,21,22,23]. In the last decade, research on spirituality at the end of life, cancer, and palliative care have increased. Likewise, research has focused on women diagnosed with breast cancer, their specific treatments, the impact of mutilating surgeries, and the emotional and spiritual factors during and after their treatment, all with well-differentiated themes and objectives. Therefore, the present study sought to give a voice to women coping with the diagnosis while valuing their spiritual needs as part of an integral (spiritual, emotional, and physical) vision and recognising that they are facing various conflicts without much time to deliberate on the decisions that should be made.

Edlund et al. [24] assert that in human beings, inner ethical dignity is found in the spiritual dimension and gives expression to the experience of dignity. It emerges from everyday life and values prevalent in a particular culture. Fundamental human rights and women’s dignity are international principles that should guide health practice in all cultural contexts [25] for an environment of dignity. Ethical principles, such as duties, rights, and responsibilities, must be respected by health professionals, who must also be conscious of integrity, honesty, and justice [26] to create an ethical environment. Active listening can also be incorporated as it is essential for supportive attention to patients’ needs [27].

Currently, health care providers who care about patients’ spiritual needs are vital in cases of breast cancer, a life-changing diagnosis that leads patients to re-evaluate their beliefs and practices and encourages them to search for transcendence [23]. Overall, holistic health care should be based on a shared understanding of spirituality in a multidisciplinary approach, with more attention paid to the person instead of the health problem, during the assessment and treatment of their emotional and spiritual distress [1,28]. However, despite patients’ right to receive treatment that considers their physical, spiritual, and emotional needs, health professionals often experience difficulties in recognising and addressing those needs [1,17]. This leads to challenges to provide more qualified and integral care.

The aforementioned literature reveals the importance of providing integral care that addresses the spiritual needs of women diagnosed with breast cancer. Therefore, the present study aimed to explore the spiritual experience of women diagnosed with breast cancer and the consideration of spirituality in health care.

## 2. Methods

### 2.1. Design

This is a qualitative study guided by Merleau-Ponty’s [29] existential phenomenology approach, which focuses on the individuals’ lifeworld. Accordingly, it is possible to comprehend individuals’ perceptive experiences by conducting phenomenological interviews. In the analysis of the qualitative data, we sought to identify patterns of common experiences shared by the participants using Amedeo Giorgi’s [30] phenomenological analysis method.

### 2.2. Setting

This study was conducted in the outpatient mastology sector of a Brazilian university hospital in the state of Rio de Janeiro, to which women with breast cancer were referred immediately after diagnosis to confirm and begin treatment. It is a high-quality, metropolitan centre in the region that attends up to 15 new cases of breast cancer per month.

### 2.3. Participants

The sampling procedure followed was intentionally based on the following inclusion criteria: women over 18 years with histopathological diagnosis of breast cancer, who had not started any kind of treatment and freely agreed to participate. Exclusion criteria included patients diagnosed with other types of neoplasms or metastasis, with more than one year of a confirmed diagnosis, or some other concomitant pathology that would make verbal communication impossible. There were no restrictions on ethnicity, religion, or economic and sociocultural political aspects.

After the breast cancer diagnosis was confirmed, participants were recruited by the researcher following a medical consultation. The recruitment took place between August 2018 and February 2019. As a preliminary step, the researcher immersed in the field of study and observed the dynamics of the sector in the setting where the study was developed. Women were observed through the breast cancer diagnosis process, including during the first medical consultation, the clinical session in which treatment decisions were made, and the follow-up nursing consultations in the pre-and post-operative periods of breast cancer.

### 2.4. Data Collection

The interviews were conducted face-to-face with the researcher in a private room for an average duration of 40 min. The characterisation form, composed of identification and sociodemographic data, was filed by the researcher (Table 1). 

A flexible and open interview script was used to promote women’s description of the world as experienced by them. The interview started spontaneously, creating a favourable environment that allowed participants to express their own experiences, feelings, and spiritual issues freely since receiving a breast cancer diagnosis. The researcher aimed to provide sensitive listening and emotional support during the session.

Each interview opened with the questions ‘what does spirituality mean to you? how do you express your faith and spiritual needs’? As the interview progressed, the themes became more profound (e.g., ‘has the diagnosis of the disease influenced your spiritual life’? and ‘do your personal beliefs influence your will to live’?) and participants were asked to elaborate and provide details. None of the participants withdrew from the study. Data collection was completed using the data saturation criterion through the repetition of information in the statements, totalling 40 interviews.

### 2.5. Ethical Considerations

This study was performed in accordance with the principles of the Declaration of Helsinki. Approval was granted by the University Research Ethics Committee (no. 64110617.20000.5243). Only those who agreed to participate in the study were included and signed an informed consent form. All study participants were given verbal and written information related to the study aims and their involvement. It was made clear that they could withdraw from the study at any time. Anonymity and confidentiality were ensured by safeguarding the data and the participants’ names.

### 2.6. Analysis and Rigour

Data analysis was supported by the MAXQDA^®^ 2018 software as an instrumental part of the analysis strategy. To accomplish transparency, the researchers discussed the study’s challenges and difficulties [27]. All interviews were audio recorded and transcribed verbatim. The data were analysed and grouped into themes to enhance comprehension of the phenomenon by three researchers.

Giorgi’s [30] phenomenological analysis method was used in a four-step procedure. First, the complete interviews were read to gain a sense of the overall meaning. Second, interviews were reread to evaluate the discrimination of meaning units, as evidenced, and to discover, articulate, and explain their psychological value and significance, with a focus on the experienced phenomenon from a health care perspective. Third, the meaning units were examined and transformed into descriptive language with an emphasis on the individual characteristics of the phenomenon. Fourth, the meaning units were synthesised to capture the essence of the experience under investigation [31,32].

To ensure the reliability of the study, our procedure followed the guidelines proposed by Graneheim and Lundman [33] and Guba and Lincoln [34]. First, the research experience was accredited in the object of the study and the qualitative methodology of the authors. Second, the researchers paid attention to their own values and beliefs, reflecting on how they could influence the different stages of the study. Third, the recruitment process with a broad selection criteria and no restrictions based on beliefs, ethnicities, ages, or other sociodemographic variables guaranteed the maximum degree of diversity in the analysed group. Fourth, data were collected through interviews conducted by the same researcher, unknown to the participants as they did not interact until that moment. Fifth, during the data analysis, the units of meaning, themes, and subthemes were differentiated, and the theoretical saturation of the main topics was verified. Lastly, we used the qualitative analysis software that allows the accreditation and documentation of the analysis as well as codification of the process. Moreover, two external researchers confirmed this analysis, according to the researcher triangulation strategy.

## 3. Results

### 3.1. Participants’ Characteristics and Identified Themes

Table 1 shows participants’ sociodemographic characteristics. The majority of women professed a religious belief (97.5%) and identified as Black, White, or Brown, with similar proportions across races. Almost half were married (45%) and had one to two children (77.5%) or more (15%).

During the analysis of the interviews, five themes were identified, which emerged from the essential meaning of this phenomenon: (1) meaning of spirituality–source of spiritual strength; (2) well-being in the relationship with God; (3) well-being in religious fellowship; (4) values and purpose of life–meaning in life; and (5) spirituality as a foundation to continue (Table 2).

These principles were revealed as being essential from the perspective of the spiritual dimension of being human. The deepest needs of the participants and how to transcend distress were recorded.

### 3.2. Meaning of Spirituality–Source of Spiritual Strength

During the interviews, the meaning of spirituality was brought up. For women diagnosed with breast cancer, spirituality was a source of support and well-being that allowed them to find themselves and make their diagnosis less distressing. Although several women reported experiencing negative feelings due to their breast cancer diagnosis, most expressed that, when facing the diagnosis, what they believed in became their source of strength. In this regard, they experienced feeling particularly closer to God and hopeful, which resulted in fewer negative feelings (e.g., anguish, hopelessness, despair). For instance, E40 mentioned, ‘it is God who is giving me this strength’. All interviewed women chose to seek spirituality as a source of strength, at a time of numerous conflicts and important treatment decisions.

### 3.3. Well-Being in the Relationship with God

Interestingly, participants reported that spirituality was a positive coping strategy, providing support, comfort, and hope that transcended common boundaries as well as a new vision of the future.

The search for communion with God, the transcendent and encounters through prayers made most of the women feel more spiritually supported at the moment of diagnosis, and consequently, more emotionally strengthened. As E14 revealed, she was believing and continuing because in God she ‘[sought] strength to get through this disease’. In other words, spirituality gave a new meaning to the situation and a more complete view of faith, confidence, purpose, and hope.

### 3.4. Well-Being in Religious Fellowship

Numerous participants reported that their relationships with their church community became stronger. They also revealed their need for someone to be praying, encouraging, and giving them comfort and support. Moreover, peace and the opportunity to feel part of the church fellowship created a different sense of significance for them: E24 described that ‘communion with brothers and sisters is very important. Knowing that everyone is praying, […] then I feel good, comforted’. In addition, the women diagnosed with breast cancer had the opportunity to be authentic and feel accepted despite their health issues by living positively with spiritual connections.

### 3.5. Values and Purpose in Life–Meaning in Life

Regarding searching for meaning in general, the participants tried to keep their sources of meaning in life the same as before the diagnosis, but most of them felt forced to look for other sources. They mentioned family (e.g., seeing their grandchildren grow, their children’s achievements), personal achievements (both personal and professional), and seeking new perspectives. In general, patients referred to God, family, and a strong will to live a healthy life as the main values guiding them. Moreover, many women, when assessing issues related to the importance of being alive, cared about their will to live, well-being, and quality of life as reasons to seek better care for themselves, including cancer treatment and healthy attitudes.

### 3.6. Spirituality as a Foundation to Continue

In many cases, women sought to take advantage of the foundation that they had experienced in their spirituality. This allowed them to keep calm and balanced for their own well-being from the time of their diagnosis onwards, which may result in living a somewhat normal life. As they mentioned, spiritual fortitude was a consequence of dynamic faith, redirecting the focus of their real lives, moving on, and ‘overcoming all barriers’ (E35). Also, an optimistic attitude was linked to hope and faith and was deemed favourable at the difficult moment of the diagnosis of breast cancer.

In confronting the disease, some women redirected their focus to spirituality, realising their capacity to positively reformulate their life purpose and maintain equilibrium with courage and confidence. Spirituality became evident as a foundation and motivating force for women to move forward, sustaining their conflictive experience with the diagnosis of cancer; as E39 said, ‘my spirituality sustains me’. Additionally, most women sought to develop their inner strength by showing faith and trust, reaffirming their life principles.

## 4. Discussion

Spirituality was found to be an important resource for women with breast cancer. Broadly speaking, spirituality has been the dimension that provides comfort and inner peace for women with breast cancer, as a universal experience, but it is also unique to the individual and has a dynamic subjective character [14,35]. Furthermore, Swinton et al. [14] revealed that spirituality encouraged hope and a new vision of the future. For most women, a relationship with God sustained the belief that everything can be changed for the better.

The reformulation of a new positive perspective proved to be very important for the participants in finding new meaning in the face of their disease, focusing on the transcendental aspect, or trying to see their best, and raising the level of spiritual well-being, as confirmed by Bovero et al. [13]. The well-being and quality of life of cancer patients are possibly based on their spirituality. In the present study, faith and trust in God appeared as providers of physical, functional, spiritual, and emotional well-being. Several studies have confirmed this important connection [1,17,23,36,37].

In the present study, there was a predominance of women with breast cancer who have religious beliefs, which was also evidenced by Gall et al. [8] and Thuné-Boyle et al. [23]. Distress caused by facing cancer may promote a search for spirituality as a path to greater spiritual well-being. Lee et al. [7], in their study of 198 Latin women in the pre-chemotherapy phase, concluded that patients with greater spiritual well-being had less anxiety and depression. Castillo et al. [12] pointed out that most of the women in their research reported trusted spirituality/religiosity as an aid when the disease was diagnosed. The women in their study described ‘relying on religious practices, such as reading the Bible and praying, to help them when they experience the onset of negative emotions’ such as anxiety, anger, and depression. Women need to live their own experiences, have space for reflection about how they perceive the world, and recalibrate themselves, including their spirituality and religiosity [4,14,38].

In the relationship with groups of religious communities, some of the women lived their relationship with God more deeply. In this way, they lived moments in which communion with the sacred is experienced together with others, ‘sacred moments when the windows to the transcendent are clearer’ [39]. In a phenomenological study, Swinton et al. [14] pointed out that several interviewed breast cancer patients who were involved in relationships and were developing a sense of meaning reported improved interpersonal relations. Koenig [19] adds that these religious groups encourage patients to take the focus off themselves and help others. With this, they develop positive emotions and feel connected to those with whom they interact.

The participants highlighted how their values and purpose in life–meaning in life–were unveiled as worthy of being reconsidered. This resembles the fact that women using their natural and perceptual ability, search for meaning and purpose during the disease, each day that they remain in this difficult situation, appreciating their health in a profound way. Giving meaning to the disease and its existence by reframing the diagnosis was especially important when faced with the possibility of the finitude of life. Several women sought new perspectives as a way to give significance to the disease. According to Pargament [39], seeing life through the lens of spirituality transforms what would be common into something special. Even painful and unquestionably difficult experiences can be perceived in this deeper dimension, with a greater and more complete purpose.

The experience of values and moral and institutional principles related to spirituality provided an important foundation for the women interviewed. Frankl [40] states that in order to constitute an existential dimension, the person must opt for religiosity and experience it. The experiential values, both absolute and particular, are clearly outlined from the confrontation with an inevitable situation of intense suffering.

The importance of values is emphasised when considering the cultural and religious diversity in a globalised world where the dignity, human rights, and reality of each patient must be respected for an environment of dignity. Ethical, moral, and social principles must be carefully observed in clinical practice. Davoodvand et al. [26] emphasised the ethical aspects of care and respect for the patient’s moral, social, and spiritual values. Clinical care has the potential to use methods to promote reflections giving meaning to events and evoking positive experiences in the face of distress [17].

One of the aspects mentioned often by the patients in this study is the importance of family, which was also confirmed by Swinton et al. [14], who stated, ‘In the face of the trauma and uncertainty of the diagnosis of breast cancer, this woman discovers a goal to fulfil and an achievement to be achieved, in this case, taking good care of her family’. Frankl [41] states that meaning in life can be found when the person does something toward others during their lifetime. The closest meaning is related to something that someone decides to do on a temporal level, a goal to fulfil, such as taking care of family, friends, and so on. Castillo et al. [12] agree with their research referring to the family as a source of fortitude for the participants.

Some of the participants stated that redirecting their focus was what helped them cope with the disease by nourishing hope in a more resilient way. Numerous participants in this study sought to maintain their normal lives. The study by Torres et al. [42] confirms that the women did not allow cancer to consume their daily lives and were grateful to God for each day won in this process.

Although the patients desire to discuss their spirituality, many professionals are still reluctant to include it in their care [43]. A study that investigated the ethical aspects of spiritual care pointed out several conditions that provide the desire to engage in a conversation about spirituality. The authors in that study highlighted the possibility of imminent death, the prolonged duration of a serious illness, and the recent reception of the diagnosis [44]. In some cultures, neglecting the effects of religious beliefs has been seriously implicated in the decision to stop cancer treatment prematurely [45].

In the context of confrontation with the diagnosis of cancer, communication between patients and health professionals is challenging. Being attentive to the real inner purpose in the patient’s words through sensitivity and relationships and establishing a personal attitude as part of their professional role, is required of health care professionals [27]. As a form of care for women coping with breast cancer, initiatives such as improving therapeutic communication, offering counselling, encouraging existential experiences, and enhancing faith can be highlighted. It is worth mentioning the importance of promoting hope, support, and the roles of culture and beliefs [46].

## 5. Study Limitations

This study has limitations that must be considered. First, although the strategy of intentionally selecting participants is fully congruent with qualitative research, this might result in non-representative samples. In the present study, it was possible to suitably represent diversity with respect to most sociodemographic variables except for religion, specifically due to the small number of women without religious beliefs. Thus, such women are underrepresented in our sample, although we do not know to what extent because the distribution of the religious beliefs of this population is not known. Another limitation is that the research was conducted in one institution only, although the present field of research covers an important and diverse population area, as shown by the profile of the participants. Further investigations should be carried out to enable comparative studies to enhance the major supportive procedures for clinical care. In this sense, future studies might complement this type of research with an objective measure of spirituality, larger samples, and different scales/questionnaires. Specifically, a mixed-methods approach could yield novel and original findings to better understand the needs and challenges of women recently diagnosed with breast cancer.

## 6. Conclusions

The different themes unveiled in this study demonstrate a broad spectrum of meanings that make up spirituality; for certain patients, the spiritual dimension presented itself not only as a new purpose in life but also as a way to find it; for others, it was intrinsically linked to the religious context. In these cases, believing in God was revealed as a source of hope, resignation, adoration, and gratitude for life.

The will to live is part of this search for meaning, of valuing spiritual reality as something that makes life more complete, even in the face of immense challenges. In this confrontational moment of diagnosis, human fragility is experienced, because even in the face of breast cancer, reality and hope for the future are merged in the appreciation of life and of every opportunity to reframe the achievements of one’s existence.

Spirituality is the foundation that supports the complex process of illness caused by breast cancer, which generates changes in the meanings and re-evaluations of patients’ experience, highlighting the need for the integration of values, the will to live, and the purpose of life. What is required is the awareness and reflection of the role of health professionals, including nurses, and their commitment to care for and be close to the patient, from a respectful and dignifying perspective, creating an environment of empathy. The findings contribute to existing literature in this field due to their depth and uniqueness, and they can encourage the promotion and maintenance of holistic and ethical approaches to health for the individual and society. Additionally, they propose to improve current professional practice by considering the ethical, cultural, and spiritual dimensions that involve health care.

## Figures and Tables

**Table 1:** Distribution of the sociodemographic and religious variables of the participants.

Variable	n	%
Age Group		
24–55	13	32.5
56–60	9	22.5
61–69	11	27.5
70 or more	7	17.5
Education		
Illiterate	3	7.5
Elementary (incomplete)	5	12.5
Elementary	7	17.5
Secondary	10	25
Secondary (incomplete)	3	7.5
Higher education	5	12.5
Higher education (incomplete)	6	15
Postgraduate	1	2.5
Religion		
Catholic	21	52.5
Evangelical	15	37.5
Kardecist spiritist	3	7.5
No religion	1	2.5
Has Income		
Yes	35	87.5
No	5	12.5
Marital Status		
Single	9	22.5
Married	18	45
Divorced	9	22.5
Widow	4	10
Race		
Brown	14	35
White	14	35
Black	12	30
Number of children		
0	3	7.5
1–2	31	77.5
3 or more	6	15

**Table 2:** Themes and statements of the participants.

Themes	Units of Significance	Statements
Meaning of spirituality–source of spiritual strength	Meaning of spirituality Spiritual strength	‘My greatest treasure is Jesus because if I didn’t have Him, I wouldn’t have this foundation to talk to, I would despair ‘I’m going to die, and now’?. E 27 ‘It seems that God is giving me such strength and that I had none of my own. It was impressive for me; it is God who is giving me this strength’. E 40 ‘I think what would give me strength now is to get even more attached to God. I feel that He is supporting me, I come calmly’. E 39 ‘It seems that I was already prepared for this, it seemed that God was on my side giving me strength. I just received the diagnosis, went home, showered, put on my uniform, and went to work’. E 34
Well-being in the relationship with God	Prayer Believing and continuing Faith and confidence	‘For now, it’s just me, I talk to God. I pray normally, I talk to Him, I ask Him to heal me, to get this disease out of my body, for Him to take care of my daughter’. E 28 ‘Prayer, I constantly talk to God and believe that He can change this situation’. E 24 ‘It is trusting in God, a superior being, I believe that He does not give us a burden that we cannot bear. I seek strength to get through this disease’. E 14 ‘I have faith in myself and I will get over it if God wants, I will get over it. [...] I trust God. He always helped me. I never fell into despair, I never got down, I’m always happy’. E 40
Well-being in religious fellowship	Spiritual comfort	‘Being there in the church is something that strengthens me, that keeps me confident in life because I have this feeling of spiritual reception that is something strong for me’. E 4 ‘Communion with the brothers and sisters is very important. Knowing that everyone is praying, we get there and talk, then I feel good, comforted’. E 24
Values and purpose of life–meaning in life	Values: God, family, and will to live a healthy life	‘My son, my family, who below God are my foundation’. E 27 ‘So, first of all, God and then our family who are always there helping us and giving us strength so that we can continue to live and seek health’. E 11 ‘I have many dreams and many projects; I want to see the things that I want to accomplish’. E 13 ‘My children and my granddaughter, but the strength comes only from God. To continue with my husband, we plan many things, we dream of doing them’. E 19 ‘The desire to live healthily is huge, my will to live is everything in my life, taking care of myself and being healthy’. E 35
Spirituality as a foundation to continue	Spiritual fortitude Courage and confidence	‘My God is bigger than anything, I have faith that I will overcome all the barriers’. E 35 ‘I am calm, we have to accept things, I trust God a lot’. E 7 ‘So, I believe that my spirituality sustains me, you know’? E 39 ‘I have always had faith; I have always believed in God. Today I accept more, [...] I can’t give up’. E 26 ‘I believe in God, I have a lot of faith, I ask for wisdom, I ask for discernment, to show me the way, [...] to help me’. E 20

## Data Availability

The data presented in this study are available on reasonable request from the corresponding author. The data are not publicly available due to privacy restrictions.
